# High-throughput phenotyping of lateral expansion and regrowth of spaced *Lolium perenne* plants using on-field image analysis

**DOI:** 10.1186/s13007-016-0132-8

**Published:** 2016-06-10

**Authors:** Peter Lootens, Tom Ruttink, Antje Rohde, Didier Combes, Philippe Barre, Isabel Roldán-Ruiz

**Affiliations:** Plant Sciences Unit - Growth and Development, ILVO, Caritasstraat 39, 9090 Melle, Belgium; INRA – UR4 P3F, BP 6, 86600 Lusignan, France; Bayer CropScience, Technologiepark 38, 9052 Ghent, Belgium

**Keywords:** *Lolium perenne*, Field phenotyping, Image analysis, Growth, Regrowth, Tillering

## Abstract

**Background:**

Genetic studies and breeding of agricultural crops frequently involve phenotypic characterization of large collections of genotypes grown in field conditions. These evaluations are typically based on visual observations and manual (destructive) measurements. Robust image capture and analysis procedures that allow phenotyping large collections of genotypes in time series during developmental phases represent a clear advantage as they allow non-destructive monitoring of plant growth and performance. A *L. perenne* germplasm panel including wild accessions, breeding material and commercial varieties has been used to develop a low-cost, high-throughput phenotyping tool for determining plant growth based on images of individual plants during two consecutive growing seasons. Further we have determined the correlation between image analysis-based estimates of the plant’s base area and the capacity to regrow after cutting, with manual counts of tiller number and measurements of leaf growth 2 weeks after cutting, respectively. When working with field-grown plants, image acquisition and image segmentation are particularly challenging as outdoor light conditions vary throughout the day and the season, and variable soil colours hamper the delineation of the object of interest in the image. Therefore we have used several segmentation methods including colour-, texture- and edge-based approaches, and factors derived after a fast Fourier transformation. The performance of the procedure developed has been analysed in terms of effectiveness across different environmental conditions and time points in the season.

**Results:**

The procedure developed was able to analyse correctly 77.2 % of the 24,048 top view images processed. High correlations were found between plant’s base area (image analysis-based) and tiller number (manual measurement) and between regrowth after cutting (image analysis-based) and leaf growth 2 weeks after cutting (manual measurement), with r values up to 0.792 and 0.824, respectively. Nevertheless, these relations depend on the origin of the plant material (forage breeding lines, current forage varieties, current turf varieties, and wild accessions) and the period in the season.

**Conclusions:**

The image-derived parameters presented here deliver reliable, objective data, complementary to the breeders’ scores, and are useful for genetic studies. Furthermore, large variation was shown among genotypes for the parameters investigated.

**Electronic supplementary material:**

The online version of this article (doi:10.1186/s13007-016-0132-8) contains supplementary material, which is available to authorized users.

## Background

*Lolium perenne* (perennial ryegrass) is a dominant species of sown grasslands in temperate regions because of its excellent forage quality [11], and is also a primary turf species with rapid growth and establishment [22, 33]. For both applications, forage and turf, the perennial ryegrass plants are cut repeatedly throughout the season and need to resume growth from existing tillers and form new ones. Understanding these two processes, leaf growth and lateral expansion through the formation of new tillers is therefore relevant to breed for optimal sward establishment, growth, tillering and persistence.

It is common practice during the first stages of perennial ryegrass breeding to evaluate large collections of genotypes as spaced plants in the field [11, 28]. Destructive measurements at several moments throughout the season are combined with visual categorical scores of growth, regrowth and rust infection to select elite plants. Such evaluation methods are inexpensive in terms of investments, but can be time-consuming, do not provide detailed information and, in the case of visual scorings, are prone to subjectivity. For example, regrowth is usually evaluated by visual inspection of the plants a few days or weeks after mowing, without any reference to the status of the plant just before or after cutting. It is therefore usually not known whether a good score is due to a high capacity to resume growth from tillers already formed before cutting, or by the formation of new tillers in the periphery of the plant. Because these two processes might be controlled by different genetic factors, a clearer differentiation would allow quicker genetic progress. Furthermore full exploitation of molecular tools to advance genetic improvement of perennial ryegrass depends on the availability of detailed phenotypic evaluation data [12]. For this purpose, methodologies that allow a higher level of resolution and precision in the determination of growth-related characteristics are required.

Recent advances in image analysis-based methods allow phenotyping large collections of plants in an objective, non-invasive way [30], enabling dynamic measurements of plant growth and development. While the use of automatic phenotyping platforms suitable for the evaluation of plants in growth chambers or greenhouses has become common practice [9, 30], these systems are particularly suited for the screening of young plants in experiments of short duration (weeks to months) [5, 14]. Linking results of evaluations carried out in indoor facilities and the behaviour of plants under field conditions is challenging due to differences in environmental factors, soil characteristics, soil volume, etc. [9]. Thus, field evaluation of crops has clear advantages [2]. This is of particular importance in perennial species, such as *L. perenne*, for which it can be relevant to evaluate growth-related parameters during a full growing season or even over several seasons [30]. In recent years spectacular progress has been achieved in the development of phenotyping methodologies that make use of image analysis to evaluate crop performance in the field [1]. However, the application of these methods to *L. perenne* and related species is rather limited as of today. For example, field-based image analysis has been used to determine ground cover in turf grasses (e.g., in bermudagrass overseeded with perennial ryegrass [10, 26]. More recently, Hunt et al. [12] described a methodology for the acquisition and processing of outdoor images to estimate dry matter of spaced, 4-month-old perennial ryegrass plants. The image analysis algorithm developed was based on colour segmentation, allowing efficient discrimination between green foliage and brown soil. The performance of this algorithm with older plants, recently cut plants containing brownish sections or photographed in different seasons of the year with varying background (soil) colour was not investigated. Such a methodology is, however, required if the purpose is to estimate lateral expansion and the capacity to regrow after cutting, as this implies comparison of images of the same plant taken at different time points [12].

It is challenging to optimize and automatize an image analysis procedure, if image acquisition and image segmentation should be able to cope with plants of different ages and images acquired at different dates under varying climates. Outdoor light conditions vary throughout the day and the season. In addition, moisture level and weed or algal growth affect soil colours, and make the delineation of the object of interest in the image difficult. Furthermore, ryegrass plants can display sectors with a yellow–brown colour just after cutting or after a rainy period, which are difficult to discriminate from the soil.

Standardization of light conditions can be achieved by one of the following options: (i) avoiding external light by photographing the plants during the night using flashes; (ii) using covers to eliminate or stabilize natural light, in combination with flashes; (iii) using a NIR (Near Infrared) camera instead of a digital single lens reflex camera (DSLR); and (iv) photographing in open air using flashes to partly stabilize the white balance. Options *i* and *ii* should render relatively uniform series of images in terms of exposure and colour temperature, which are ideal for colour-based segmentation methods. While option *i* is simpler, it can be logistically difficult. Option *ii* requires the use of a mobile construction to cover the plants for photographing (see for example [3, 12]), making it rather impractical when large plants are photographed. With regards to option *iii*, commercially available NIR cameras have a relatively low resolution and are more expensive than DSLR cameras. Therefore, option *iv* is currently the most straightforward to implement. This choice has implications for image processing and segmentation because colour segmentation alone cannot be used due to unstable light conditions [17, 27].

The aim of image segmentation is to partition the image into regions that are distinct from each other, but internally uniform with respect to certain properties [17], allowing to separate the plant from the background. Segmentation methods can be divided into several types [8, 19, 23] of which colour-based, texture-based, edge detection-based and fast Fourier transformation are frequently used. Colour-based segmentation, based on colour differences between plant and background, can make use of different colour spaces but in horticulture and agriculture RGB and HSV spaces are commonly used [20]. RGB is the standard colour space used by the detector of DSLR cameras but it does not correspond to the way humans see colour [21]. The HSV space is, for human interpretation, more convenient. As the Hue channel (H) is relatively invariant to light level and shading, segmentation can be performed on the H dimension alone [31]. An alternative is to normalise the intensity levels of a colour channel using another colour channel (e.g. using the ratio of B/G in the RGB colour space) [7] or apply other transformations [20]. Although commonly used, colour-based segmentation methods might be less effective to process images taken outdoors. First, the parameters used for the colour segmentation are very sensitive to the white balance, which is affected by sun conditions (under cloudy conditions colours are perceived differently than under sunny conditions by imaging sensors). Second, although automatic light measurement by the camera generates images of the same quality (light, colour), the effective amount of light captured depends on the scene [20]. Third, light reflection on waxy leaves can result in bright or even white spots in the image, and corresponding colour values need to be included in the colour range for selection. Fourth, the presence of algal growth or weeds may hamper the correct identification of the object of interest because they are also green. Texture-based segmentation methods make use of a variance operator to identify regions with different textures (different repeated pattern of different pixel intensities), enabling the separation of objects with the same colour but different textures (for example soil covered by green algae and a green grass plant, or between brownish soil and brown plant parts) [21]. However, segmentation based on texture will be hard for thin-leaved, small plants compared to large plants displaying a more uniform texture. This problem is less pronounced in edge-based segmentation methods, which identify regions in the image where brightness changes rapidly. Ideally edge detection leads to a set of connected curves that correspond to the boundaries of the object [15]. Because this method is rather sensitive to noise, resulting in the detection of irrelevant features in the image, a Gaussian filter is usually applied before the edge detection procedure is executed [4]. Colour-, texture- and edge-based segmentation methods make use of the spatial domain of an image. Alternatively, an image can be converted by fast Fourier transformation to the so-called frequency domain showing frequency and orientation. Erasing information from a location in the frequency transformation is equivalent to removing the corresponding information in every part of the spatial domain image [18, 21]. As a result, the rough plant contours can be selected as the main feature of an image.

From the characteristics of the different segmentation approaches summarized above, it is clear that combining information generated by different methods can result in a more accurate segmentation that exploits their complementarities. It follows that a considerable improvement in segmentation can result from the combination of colour, texture, and edge information [13, 17].

Here, we present a method for high-throughput phenotyping of lateral expansion and regrowth of spaced *L. perenne* plants based on images taken in the field using a DSLR camera placed on a tripod. During image processing, information derived from different segmentation methods is compared and then combined via post-processing integration enabling robust plant object recognition. We show that the combined approach renders better results than the different segmentation methods separately and that the methodology developed is effective under different illumination conditions in outdoor environments. Based on top view images taken at specific time points, the area covered by the *L. perenne* plants was determined in a standardized, quantitative way. We show high correlations between image analysis-based estimates of plant growth and manual measurements.

## Methods

### Plant material and field trial description

A total of 501 genotypes constituting a diverse genetic and morphological collection were planted in a nursery in Melle, Belgium (N50°59′32″ E3°46′59″). Genotypes from four main sources were considered: ‘forage breeding lines’ (n = 117), ‘current forage varieties’ (n = 50), ‘current turf varieties’ (n = 69) and ‘wild accessions’ (n = 265). ‘Forage breeding lines’ comprises genotypes from different European breeding programs, ‘current forage varieties’ and ‘current turf varieties’ comprise genotypes from commercial varieties, and ‘wild accessions’ comprises genotypes from natural accessions originating from across Europe. Individual plants containing 3–5 tillers and trimmed to 5 cm were planted at 75 cm spacing within and between rows in a randomised block design with three blocks and one clonal replicate per genotype per block. The field was established on October 2009, and was maintained for three consecutive years with regular weeding and fertilisation. The plants were cut at 6-week intervals during 2010 (March 17th (Y1C1), May 4th (Y1C2), June 15th (Y1C3), July 29th (Y1C4), September 6th (Y1C5) and October 26th (Y1C6) (Additional file 1: Fig. S1). During 2011 all plants were cut on March 16th (Y2C1); in the period May–June 2011 individual plants were cut around 3 weeks after their respective heading date (Y2C2), and all plants were again cut at the same day on July 14th (Y2C3), and August 29th (Y2C4).

### Image acquisition

**Fig. 1:**
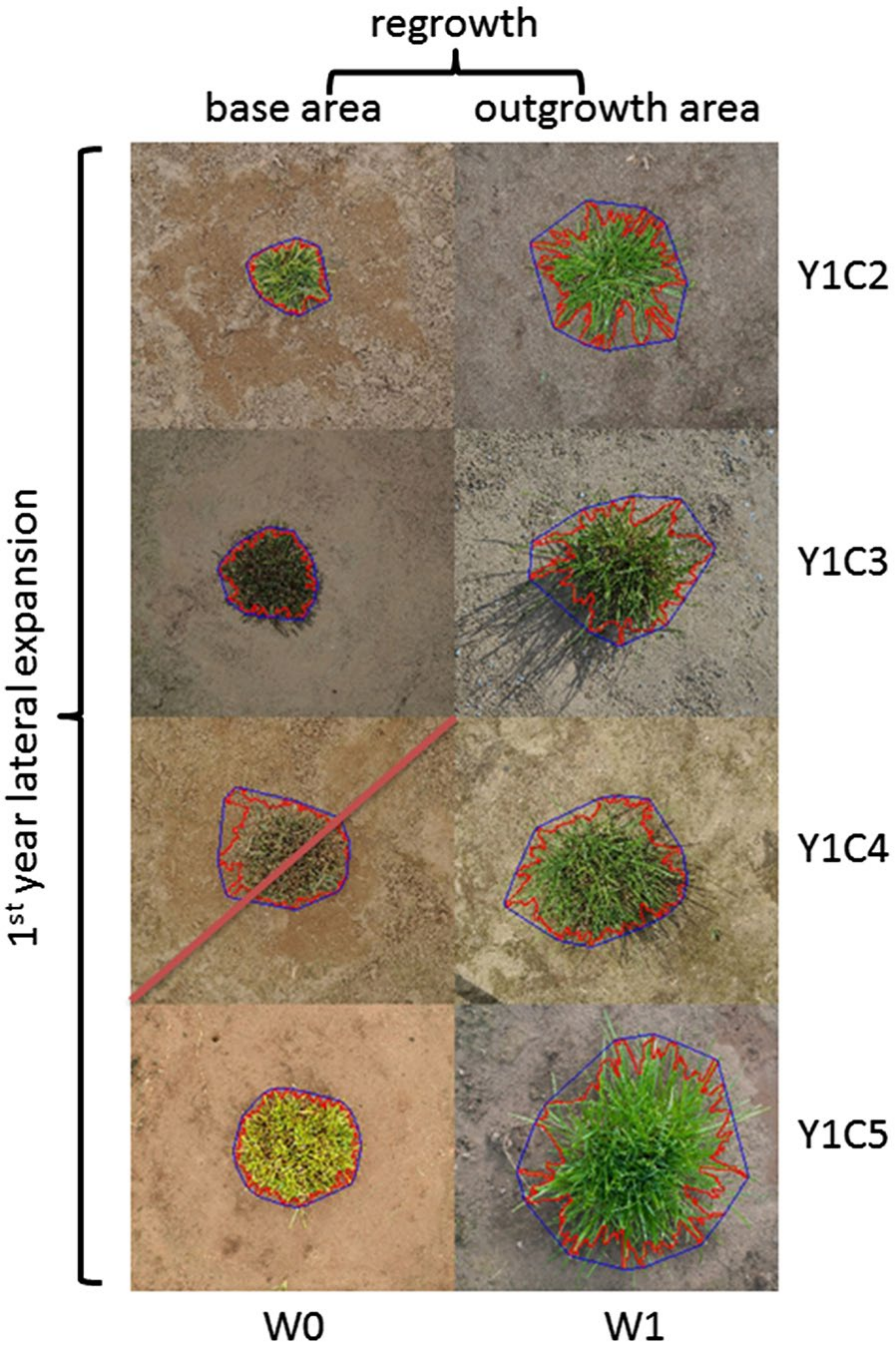
Top view images of the same *L. perenne* plant. Plants were cut every 6 weeks in Year 1. Immediately after the cut an image was taken showing the base area of the plant (W0, week 0), after 1 week of regrowth a second image was taken showing the outgrowth area (W1, week 1). The selected plant (*red outline*) and convex hull (*blue outline*) found by the image analysis algorithm are superimposed on the images. Based on these selections plant variables are calculated. Here, one image (Y1C4W0) was not correctly analysed and the derived data was not used

Top view images were taken from each individual plant directly after cutting (W0) and 1 week after (W1) for the time points Y1C2 to Y1C6 and Y2C1, Y2C3 and Y2C4 (Fig. 1; Additional file 1: Fig. S1). Images were acquired using a DSLR camera (D90, Nikon Corporation, Japan) with a 35 mm lens (AF-S NIKKOR 35 mm 1:1.8G, Nikon Corporation, Japan) or a 24 mm lens (AF-S NIKKOR 24 mm 1:2.8D, Nikon Corporation, Japan) and three wireless remote speedlights (SB-R200, Nikon Corporation, Japan) controlled with a wireless commander (SU-800, Nikon Corporation, Japan). The images for the first three time points (Y1C2, Y1C3 and Y1C4) were saved as JPG files. For the later dates also NEF (raw) images were recorded, as this offers more possibilities for correction of the white balance, exposure or contrast without data loss. A tripod (055XPROB + 804RC2, Manfrotto, Italy) was used, with the camera placed in perpendicular orientation with respect to the soil surface. The distance between the camera objective and the soil was 90 cm. Images were taken using the Live View function of the camera for the ease and speed of working. Before taking images the white balance of the camera was adjusted to the weather conditions (cloudy or sunny), and an image was taken of a reference card (Grey card, Novoflex) to transform from pixel-scale dimensions to cm-scale dimensions, and to have a colour-neutral reference. The resolution of the images was 74.36 pixels per cm at soil surface when using the 35 mm lens and 49.57 pixels per cm when using the 24 mm lens.

### Image analysis procedure

To optimise the image analysis procedure we chose a subset of 25 genotypes (in three replicates, 75 plants) that represent the broad range of phenotypic diversity (such as base area after cutting, tiller number, plant height and increase in leaf length after cutting) present in the plant collection of 501 genotypes. This subset of 1200 images, representing clonal replicates and different time points of these 25 genotypes, covered various aspects of variation present in the complete set (501 genotypes and 24,048 images), ranging from light spectral quality and intensity, to plant size and colour, and variation in soil background conditions (dry, wet, algae coverage present, small weeds present). Once an optimised and validated procedure was established, it was applied on the total of 24,048 images of all plants and time points.

#### Overall description

The images were processed using an automatic program developed in WiT (8.3 sp7, Dalsa Digital Imaging Inc., Canada) (Additional file 2: Fig. S2). Each image was segmented using eight segmentation methods available in WiT (here called C1, C2, T1, T2, E1, E2, E3 and E4) as described below. In addition, based on the resulting mask for each segmentation method, a composite mask image was constructed in which each binary mask had a weight of 1/8th of a maximum intensity of 255. For this composite mask image the pixel intensity threshold was set at 128. This means that in the composite mask, a pixel belongs to the object (i.e., the ryegrass plant) if it belongs to the masks derived from at least four (out of eight) methods [256 (possible intensities) divided by 8 (methods) multiplied by 4 (correct methods) = 128]. For the resulting composite mask only the largest object was kept and used to produce an object outline overlay on the original image, depicting the edge of the final selection. This image was stored. After all images had been processed automatically, we determined by visual inspection if the automatic delineation of the ryegrass plant was correct. The images where parts of the plants were not detected or mismatched were not used for the between-method comparisons described below. Examples are shown in Fig. 1.

#### Segmentation methods

An overall scheme of the image analysis procedure is shown in Additional file 2: Fig. S2. Eight different segmentation methods were combined. These include colour-based methods (C1, C2), texture-based methods (T1, T2) and edge based-methods (E1–E4) (Table 1). Intensity values for the HSV colour space used within WiT range from 0 to 255 for all channels.

**Table 1:** Description of the different segmentation methods [colour-based (C); texture-based (T); edge-based (E)] used during the images analysis of top view images of *L. perenne* plants

Name	Segmentation method	Colour space—channel	Domain	Method (WiT)	Extra tools used	
					Before	After
C1	Colour threshold	HSV–HSV	Spatial	Threshold		Dilate, fill holes, erode, selection of the largest object
C2	Colour threshold	RGB-B/G	Spatial	Threshold		Dilate, fill holes, erode, selection of the largest object
T1	Texture	HSV-S	Frequency	Fast Fourier transform and cosine filter		Dilate, fill holes, erode, selection of the largest object
T2	Texture	HSV-S	Spatial	Entropy measurement		Dilate, fill holes, erode, selection of the largest object
E1	Edge	HSV-S	Spatial	Gradient magnitude and direction filter (Prewitt)	Gauss filter	Dilate, fill holes, erode, selection of the largest object
E2	Edge	HSV-S	Spatial	Two dimensional convolution	Gauss filter	Dilate, fill holes, erode, selection of the largest object
E3	Edge	HSV-S	Spatial	Prewitt edge detection	Gauss filter	Dilate, fill holes, erode, selection of the largest object
E4	Edge	HSV-S	Spatial	Refine edges based on Prewitt	Gauss filter	Dilate, fill holes, erode, selection of the largest object

For all segmentation methods except C2 the RGB image was converted to an HSV colour space to enable an easier selection of the green colour: greenish leaves can easily be found between intensities of 35 and 100 of the Hue (H) colour channel. We used threshold boundaries for the H, S and V colour channels of 35–100, 50–255 and 40–226, respectively. The resulting image, which is a raw mask, was then cleaned using a dilate-erode process to remove small objects not related to the plant, and the holes within this mask were filled. This procedure of cleaning up the raw mask was the same for all methods.

For C2 the RGB image was used, and the ratio between the blue (B) and the green (G) colour channel was calculated. In this ratio image the plant appears with a lower intensity than the background. A threshold of 0.625 was used to select the plant object.

The calculations for the texture and edge based methods (T1, T2 and E1–E4) were all based on the saturation (S) channel of the HSV colour space. This colour channel was chosen because of its high contrast between background and object. In T1 a fast Fourier transform operator was used to calculate a frequency domain image. After using a cosine filter with a radius of 25, a reverse fast Fourier transform was calculated resulting in a spatial domain image. The threshold was set at 110. Intensities above this value were used to delineate the raw mask. T2 makes use of an entropy operator. This operator calculates the entropy of the input image pixel values in a specified neighbourhood (100 × 100) around each pixel. In this context, the entropy value of a pixel is a measure of the disorder in the neighbouring pixels. High intensity changes within a limited area due to the transition from plant to the background results in an entropy image in which the plant gets a higher intensity than the background. The threshold was set at 4.7.

For the edge-based methods, E1–E4, a Gaussian filter with a filter size of 5 × 5 was used first to achieve a two-dimensional smoothing of the input image. This avoided the selection of irrelevant edges. E1 used a gradient magnitude and direction filter based on Prewitt filter weights. A threshold was set at 29. E2 used a two-dimensional convolution operator with a 3 × 3 kernel. The elements in the kernel were set to 1 except for the middle kernel value, which was set to 15. A scale factor by which all values in the convolved image are divided was set to 13. The threshold was set at an intensity of 230. For E3 an edge-detect operator was used to detect areas of high slope using a Prewitt gradient type operation. The threshold was set at 27. Finally, E4 used a refine edges operator based on a Prewitt kernel with a threshold set at 15. On the resulting image of the raw mask a threshold intensity of 1 was applied.

The parameter settings for the different methods, as described above, were determined using a selected subset of 40 images taken at different time points throughout two subsequent growing seasons, representing different light colour (depending on the time of the day that the images are taken), different background colour or texture, different plant size, etc. Histograms of sections of the images belonging to the plant or the background were extracted. Based on inspection lines showing local intensities, and based on visual interpretation of the image, values were optimised in an iterative process. Based on the visual inspection of the results of each method and the composite mask (combining information from all 8 methods), overlaid on the original image (Fig. 1), the success rate per time point was determined. Based on these results, the threshold settings of the different segmentation methods were refined. Finally, an optimum was found so that a maximum number of images was correctly analysed and with similar success rates for all time points. In the final analysis for the optimisation stage these optimal settings were used for all images (1200 in total).

#### Evaluation and comparison of segmentation methods

The performance of the eight segmentation methods was evaluated according to the methodology described by Van Rijsbergen [29] and Smochina [25]. Precision (*P*), Recall (*R*), and the harmonic mean of P and R (F value, denoted as parameter F = 2PR/(P + R)) were determined. The parameter *P* estimates how many pixels in the mask of the corresponding segmentation method do really belong to the object of interest. The parameter *R* estimates how many pixels of the object (i.e. plant) are included in the mask.

Comparisons using these three parameters were carried out at two levels. First, the agreement between methods was evaluated in a pairwise fashion by using the mask generated by one of the methods as ground-truth and calculating P, R, and F for the mask generated by the other method. In total 28 comparisons (8 × 7/2) were made per image, and all 1200 images are used for this analysis. Second, we selected by visual inspection a subset of images in which the overlay of the composite mask (see above) fitted precisely on the plant (Fig. 1). For these images, the image analysis procedure accurately outlined the plant object, and we used the composite mask as the ground-truth. The eight different segmentation methods were then compared to that composite mask to establish the correctness of each method. Note that because only for the correctly analysed images a ground-truth area (i.e. composite mask) was available, the correctness of the methods is slightly overestimated.

### Image analysis-based parameters

Once the image analysis procedure had been set up, the images of 501 genotypes and all time points (24,048 images in total) were processed in an automatic way. For each correctly processed image, the number of pixels contained in the composite mask corresponds to the area covered by the plant either directly after cutting (termed ‘base area’; W0), or 1 week later (termed ‘outgrowth area’; W1). Pixel data were converted to cm^2^. The short term regrowth after cutting, here termed ‘regrowth’, was calculated as outgrowth area minus base area at a given cut (e.g. Y1C2W1–Y1C2W0, Fig. 1).

### Manual measurements

We counted the number of tillers of each individual plant prior to each cut (in what follows ‘tiller number’) and recorded the plant height 2 weeks after each cut by measuring with a ruler the length of the longest leaf when stretched vertically. Plant height data were used to calculate leaf growth per growing degree day for each cut (in what follows ‘leaf growth’). These measurements were only carried out in Year 1. Minimum and maximum daily temperatures were measured in a weather station within a distance of 500 m from the field trial. A base temperature of 0 °C was used for the calculation of thermal time.

### Data analysis

To estimate the correspondence and complementarity between image analysis-derived data and manual measurements we compared sets of data as follows. First we determined the correlation between ‘leaf growth’ over 2 weeks after cutting (manual measurement) and ‘regrowth’ (derived from image analysis). Second, we correlated ‘tiller number’ (manual measurement) and ‘base area’ (derived from image analysis).

Finally, we plotted the changes in ‘base area’ over the whole growing season and calculated the total lateral expansion during the first growing season, here termed ‘first year lateral expansion’ (Fig. 1), as the slope of the regression for the base areas (W0) of Y1C2, Y1C3, Y1C4, and Y1C5 (cm^2^ °C day^−1^). Only plants with at least three valid observations were used to estimate ‘first year lateral expansion’.

In all these calculations average values per genotype were derived from data of the three clonal replicates in the field trial. Correlations were calculated using Statistica (v12, StatSoft Inc., Tulsa, Oklahoma, US).

## Results

### Overall evaluation of the image analysis segmentation methodology

**Fig. 2:**
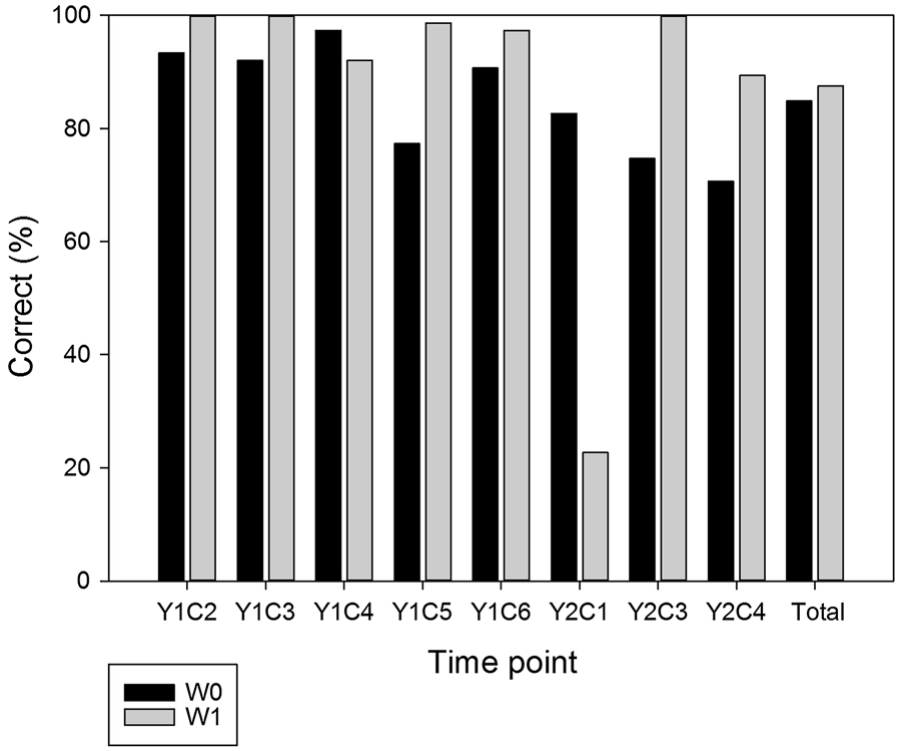
Percentage of correctly analysed images for the different time points over two growing seasons. *Y* year, *C* cut; *black bars* represent images of plants that are just cut, base area (W0); *grey bars* represent images of plants that were allowed to regrow for one week after cutting, outgrowth area (W1) (mean, n = 75)

We first used colour segmentation on the earliest series of images taken. No single segmentation method resulted in a satisfactory number of images that were correctly analysed over all series (a series refers here to a set of pictures taken at a single time point, e.g. Y1C2W0), because of the large variation in light quality and intensity, background and plant characteristics among the different series of images (data not shown). Therefore, we combined the masks of eight segmentation methods into a composite mask to improve the robustness of the overall image analysis procedure. We analysed all the images of 75 plants (corresponding to 25 genotypes) across two growing seasons. We inspected visually whether the overlay composite mask correctly outlined the actual plant form in each image and found that 86.2 % of the images were correctly analysed (Fig. 2). This shows that the combination of different segmentation methods allows a correct assessment of the images, independent of the light conditions, background characteristics, and plant size throughout two growing seasons. An exception concerns the mages of Y2C1W1, of which only 22.7 % were correctly analysed. This low success rate was because at the time of image acquisition the soil had a similar texture as the plant due to suboptimal weeding. Under these circumstances typically E4 and E1 yielded poor results. This further shows that field management is an integral part of proper image acquisition and contributes to the quality of downstream image processing.

Over all cutting periods, 84.5 % of the images of plants that were just cut (W0) were correctly analysed, while 87.5 % of the images of plants that were allowed to regrow for 1 week (W1) were correctly analysed. After elimination of Y2C1W1 data, the success rate increased from 87.5 to 96.8 %, demonstrating a higher success rate for images of plants that were allowed to regrow for 1 week and which displayed fewer brownish sectors. Indeed, after 1 week of regrowth most visible plant parts are green and are easier to delineate using image analysis (Fig. 1).

### Comparison of segmentation methods

The overlap of the masks defined by independent segmentation methods was evaluated based on all 1200 images. Per image, the resulting mask of one method was treated as ground-truth, irrespective of whether the image was correctly analysed or not, and compared to the masks defined by each of the other seven segmentation methods. Thus, per image a total of 28 pairwise comparisons were made. The average of all harmonic mean (F) values for all 1200 images was used for pairwise comparisons of methods (Table 2). The highest correspondence across all images was found between methods E1 and E3 (96.2 %), followed by E1 and E4 (92.1 %), and E3 and E4 (89.4 %). These are all edge-based. E2 displayed the lowest correspondence with all other segmentation methods (average F values between 50.6 and 59.1 %). The two colour-based methods C1 and C2 showed 77.8 % correspondence, while the texture-based methods T1 and T2 showed 66.8 % correspondence. Other combinations showed intermediate values. This clearly shows that the different methods can be complementary, even though some methods with high correspondence may be redundant.

**Table 2:** Overlap of the masks derived from eight different segmentation methods [colour-based (C); texture-based (T); edge-based (E)] described in Table 1 estimated as the average of F values (=harmonic mean of precision and recall), n = 1200

	Colour segmentation		Texture segmentation		Edge based segmentation			
	C1	C2	T1	T2	E1	E2	E3	E4
C1		77.8	65.7	72.8	74.4	50.6	75.0	71.5
C2			79.7	76.5	77.7	58.5	78.1	75.5
T1				66.8	65.9	50.9	66.4	63.3
T2					88.6	56.3	88.1	86.8
E1						57.1	96.2	92.1
E2							56.1	59.1
E3								89.4

### Performance of the different segmentation methods relative to the composite mask

Next, we compared the masks derived from the different segmentation methods to the composite mask. Since here we were primarily interested in how accurate the different methods could find the ryegrass plant in the image, we used only the 1034 images (86.2 % of the complete set) in which visual inspection confirmed that the composite mask accurately outlined the plant object.

**Fig. 3:**
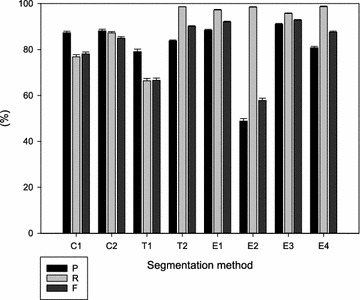
Precision (P, %), recall (R, %), and harmonic mean of precision and recall (F, %) of the different segmentation methods [colour-based (C); texture-based (T); edge-based (E)] for all time points together. The composite mask derived from eight segmentation methods was used as the ground truth in these comparisons (mean and SE, n ≤ 75)

A high Recall (R) in combination with a high Precision (P) is ideal. A high Recall in combination with a low Precision indicates a mask that overestimates the area of the ryegrass plant. The harmonic mean of P and R, also called the F value, represents the performance of object recognition. The highest F values were found for E3 (92.8 %) and E1 (92.0 %) (Fig. 3). E3 had a slightly lower R than E1 whereas E1 had a slightly lower P. This indicates that E1 overestimates the plant area more than E3. E2, another edge-detection method, had the lowest F value (57.9 %). The method clearly finds the object (high R) but overestimates its area (low P). The colour-based methods (C1 and C2) had an intermediate F value of 78.1 and 85.0 % respectively. C2 performs better than C1 in terms of R. T1, a texture-based method, displays an intermediate performance, between E2 and the colour-based methods. In comparison to E2, the R of T1 is lower but the P is higher. T2 and E4 show slightly lower F values compared to E3 and E1. Nevertheless, both show a higher R but a lower P.

**Fig. 4:**
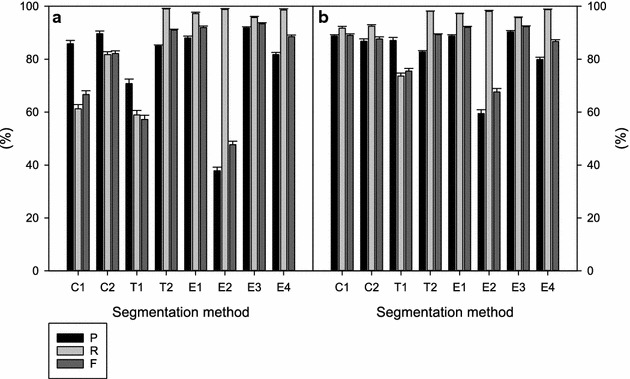
Precision (P, %), recall (R, %), and harmonic mean of precision and recall (F, %) of the different segmentation methods [colour-based (C); texture-based (T); edge-based (E)]. **a** Images of plants that were just cut (base area; W0); **b** Images of plants that have regrown for 1 week (outgrowth area; W1). The composite mask derived from eight segmentation methods was used as the ground truth in these comparisons (mean and SE, n ≤ 75)

The colour-based method C1, the texture-based method T1, and the edge-based method E2 display higher F values for W1 (Fig. 4b) than for W0 images (Fig. 4a). Segmentation methods C2, T2, E1, E3, and E4 performed in a similar way for W0 and W1 images, and were less dependent on the fine structure of the plant.

### Description of plant growth characteristics during the first year based on image analysis

A total of 24,048 images were processed using the procedure developed. For 77.2 % of these images (18,565 images) the procedure yielded correctly analysed images. Based on temporal series of images of base area (W0) and outgrowth area (W1) taken at repeated cuts during the first growing season (Y1), three different aspects of plant growth dynamics were assessed for each genotype. We present results for the whole collection of genotypes and for four subsets (forage breeding lines, current forage varieties, current turf varieties and wild accessions), expected to differ morphologically.

**Fig. 5:**
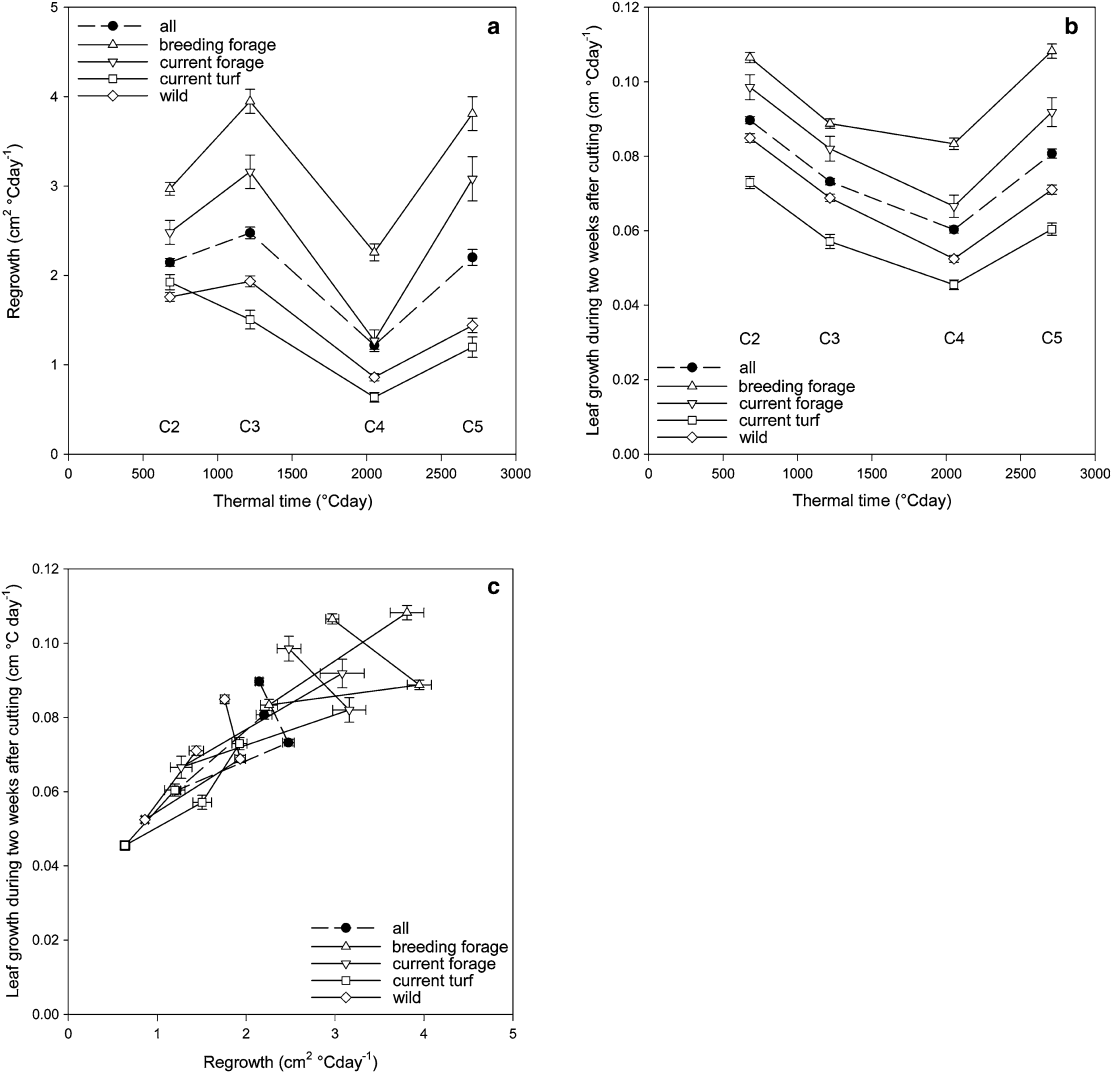
**a** Regrowth (cm^2^ °C day^−1^) and **b** leaf growth (cm °C day^−1^) versus thermal time (°C day) and **c** leaf growth (cm °C day^−1^) versus regrowth (cm^2^ °C day^−1^) for different groups of genotypes and for the total collection in Year 1 (mean and SE, n = 501)

First, by subtracting the base area (W0) from the outgrowth area at 1 week after cutting (W1) (Fig. 1), we estimated the short-term regrowth after cutting, here termed ‘regrowth’. This measurement is related to the capacity to quickly regain leaf surface and photosynthetic active biomass. ‘Regrowth’ varies throughout the season (Fig. 5a), consistent with general seasonal growth patterns and environmental constraints. Cut Y1C2 in spring and Y1C3 in early summer are characterised by relatively strong ‘regrowth’. Y1C4 represents summer growth depression after flowering and during warmer months with lower water availability. At cut Y1C5 in autumn, plants show ‘regrowth’ that is on average comparable to that in spring. As expected, at all cuts, ‘regrowth’ of forage types (breeding forage lines and current forage varieties) was higher than that of turf types and wild accessions.

**Fig. 6:**
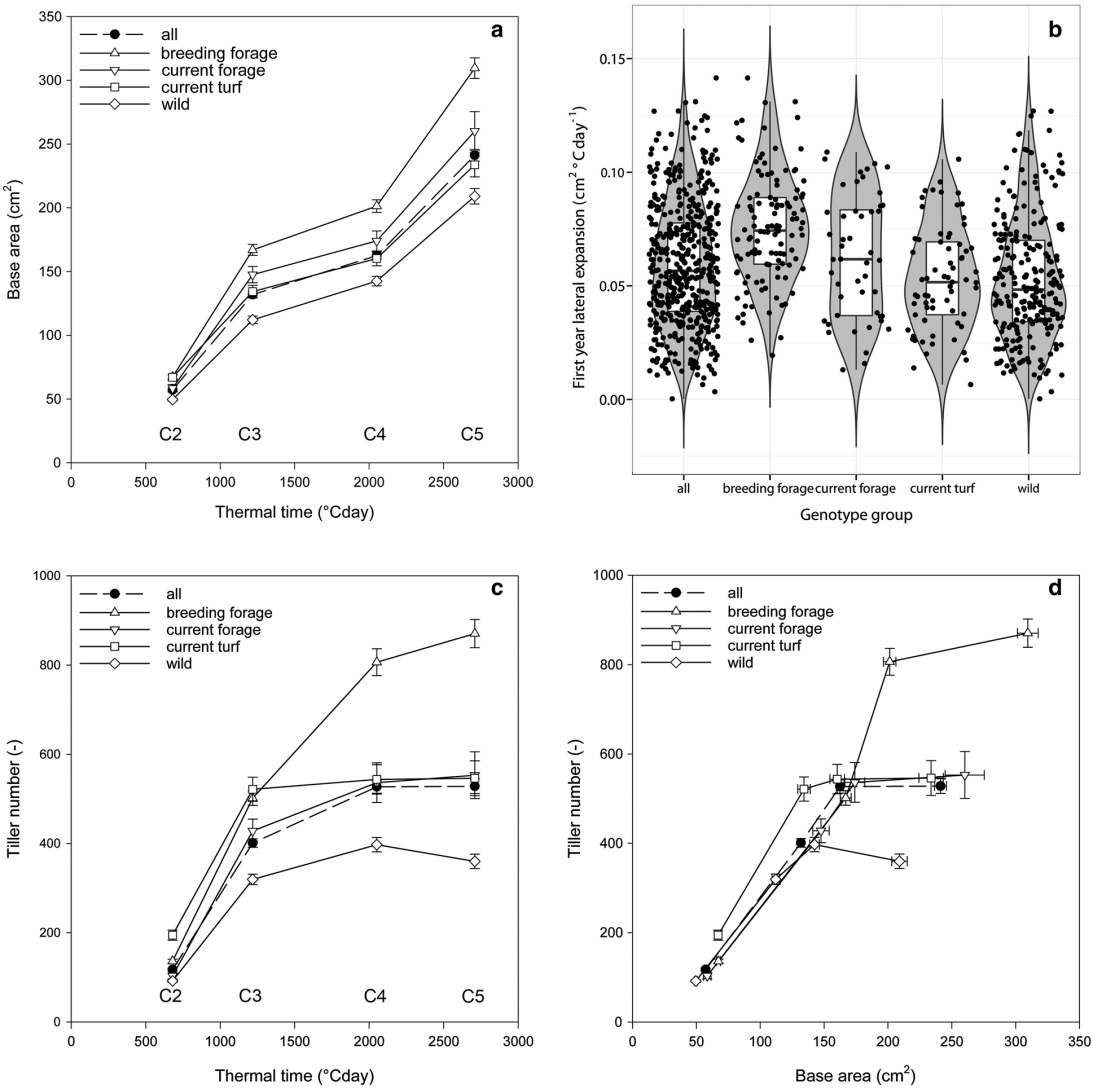
**a** Evolution of base area (cm^2^) versus thermal time (°C day^−1^), **b** first year lateral expansion (cm^2^ °C day^−1^) for different groups of genotypes and for the total collection in Year 1, **c** evolution of tiller number versus thermal time (°C day^−1^), and **d** relation between tiller number (–) and base area (cm^2^) (mean and SE, n = 501)

Second, by plotting ‘base area’ (W0) calculated for subsequent cuts in year 1, we investigated the plant growth over a whole growing season (Fig. 6a). This increase in ‘base area’ is mainly due to the production of new tillers in the periphery of the plant. Strong lateral expansion growth is observed early in the season (between Y1C2 and Y1C3), followed by a period of little increase during summer after flowering (between Y1C3 and Y1C4), and further lateral expansion in autumn (between Y1C4 and Y1C5). Also in this case, lateral expansion was higher for forage types (forage breeding lines and current forage varieties), as compared to current turf varieties and wild accessions.

Third, a linear regression of the ‘base area’ measured directly after cutting (W0) across the entire first growing season (Fig. 1) reflects the global lateral expansion growth, here termed ‘first year lateral expansion’. This is an important factor for sward closure during the first growing season. For the 501 genotypes, the first year lateral expansion was on average 0.059 ± 0.001 cm^2^ °C day^−1^ with a maximum of 0.141 cm^2^ °C day^−1^. Breeding forage lines and current forage varieties show significantly higher first year lateral expansion rates than wild accessions and current turf varieties, which is to be expected as current forage varieties are selected for productivity and sward forming capacity (Fig. 6b). It should be noted here that the cutting frequency applied in this experiment was probably not sufficient to stimulate vigorous lateral expansion in turf types, typically selected to very frequent cutting. A relatively large phenotypic variation is present in the genepool, indicating that further improvement for this trait is possible. For instance, a number of wild accessions have higher ‘first year lateral expansion’ rates than the average within forage breeding genotypes, current forage genotypes, or current turf genotypes.

### Correlation between on-field measurements and image analysis data

Next, we compared counts of ‘tiller number’ and measurements of ‘leaf growth’ on the one side with the image analysis derived parameters ‘base area’ (W0) and ‘regrowth’, respectively, to estimate whether image analysis could replace and/or yield complementary data for the evaluation of plant growth.

‘Leaf growth’ after the cut was variable throughout the season (Fig. 5b), consistent with summer depression around Y1C4 as observed in the ‘regrowth’ measurements based on image analysis (Fig. 5a). However, the estimated ‘leaf growth’ was lower at Y1C3 than at Y1C2. This was not the case for image analysis-based regrowth estimations (Fig. 5a). Probably, young plants grow initially in the vertical direction, which is not easily detected using top view images. The correlations between ‘leaf growth’ after cutting and ‘regrowth’ were 0.645, 0.608, 0.792 and 0.790, respectively, for Y1C2, Y1C3, Y1C4, and Y1C5 when all 501 genotypes were considered together (Table 3; Fig. 5c). The correlations were all statistically significant. However, the correlation values are different for the different groups and depend on the cut considered (Additional file 3: Fig. S3). This illustrates that plants that are able to resume growth quickly after cutting can be identified using image analysis, but that the two ways of measurements can reflect different aspects of growth, and are therefore at least partially complementary. This is because both, variation in the rate of outgrowth of leaves as well as variation in leaf orientation (erect or prostrate), affect the estimation of ‘regrowth’ estimates, while ‘leaf growth’ measurement concerns the longest leaf of the plant 2 weeks after cutting, irrespective of its orientation.

**Table 3:** Correlation (r) between leaf growth (cm °C day^−1^) based on height at 2 weeks after cutting and regrowth (cm^2^ °C day^−1^) determined by image analysis (top panel) and between tiller number and base area (cm^2^) determined by image analysis (bottom panel), at four consecutive cuts (between C2 and C5 of the first growing season)

Genotype groups	Y1C2	Y1C3	Y1C4	Y1C5	Y1 all cuts
*Correlation between regrowth and leaf growth*					
Forage breeding lines	0.543	0.425	*0.758*	*0.769*	0.567
Current forage varieties	*0.765*	0.508	0.685	0.523	0.626
Current turf varieties	0.735	*0.682*	0.651	0.753	0.770
Wild accessions	0.462	0.316	0.548	0.595	0.529
All groups	0.645	0.608	0.792	0.790	0.686
*Correlation between base area and tiller number*					
Forage breeding lines	0.758	0.682	0.636	0.669	0.824
Current forage varieties	*0.847*	*0.793*	0.686	*0.824*	0.799
Current turf varieties	0.769	0.576	0.461	0.609	0.638
Wild accessions	0.826	0.739	*0.726*	0.740	0.727
All	0.782	0.731	0.721	0.755	0.769

All correlations were significant (p < 0.05). The highest r values per group are depicted in italics. The highest r values for all groups or for all cuts are underlined

‘Tiller number’ increased rapidly between Y1C2 and Y1C3 (Fig. 6c). While ‘tiller number’ continued to increase throughout the first growing season in current and forage breeding lines types, it almost stabilised after Y1C3 in early summer in turf types and wild accessions. On average, wild accessions produced significantly less tillers per plant than other groups. The correlations between ‘base area’ and the ‘tiller number’ ranged between 0.721 and 0.782, with the highest correlations at Y1C2 in spring and Y1C5 in autumn (Table 3; Additional file 4: Fig. S4). This shows that ‘base area’ increased due to the formation of new tillers. The relation between the ‘base area’ (Fig. 6a) and ‘tiller number’ (Fig. 6c), remained relatively constant over all groups in the first three cuts (Y1C2–Y1C4; Fig. 6d). This relation changed late in the season (Y1C5): while the ‘base area’ kept on increasing, the increase in ‘tiller number’ became lower than earlier in the season, so that the overall tiller density decreased in autumn (Y1C5).

Taken together, our data demonstrate that image analysis provides measurements of plant growth that are relevant for the evaluation of plant performance under field conditions. These estimations are largely correlated but for some aspects are also complementary to morphological measurements. The main advantages of the image analysis procedure presented here are its objectivity and the fact that it can be applied easily to thousands of plants at repeated time points in the season.

## Discussion

### Method development

Here we present a new image analysis method to phenotype (re)growth characteristics of field-grown *L. perenne* plants. The method is robust to daily and seasonal changes in light conditions and to different background (~soil) colours and textures. When applied to a total of 24,048 images of plants that were just cut or after a short period of regrowth, and recorded at different time points throughout the season, 77.2 % of the images were correctly processed, allowing quantitative estimations of plant size (‘base area’ and ‘outgrowth area’).

Given the difficulties associated to the acquisition of images outdoor and their processing, the development of a robust methodology able to cope with differing light intensity and spectrum and with different backgrounds is not a simple task. In our experience, one operator can take a series of 500 images in about 2 h, which allows photographing a few thousand plants on a weekly basis. While images of a whole field can be captured in only a few hours, still light conditions (spectral quality and intensity) may change during this period. We suggest using speedlights and semi-automated settings on the camera: a minimum diaphragm opening to have sufficient depth of field and a minimum shutter speed, which equals the maximum synchronisation time of the flashes. For image processing here we combine the power of eight segmentation methods in a highly automatized way. Further improvements are however possible at the level automation of the image analysis step with online extraction of parameters. Furthermore, given the high correlation detected for some pair-wise comparisons among the eight methods tested here, a subset of segmentation methods could possibly be chosen. By preference, the methods with the highest Precision (P) and Recall (R), which are fast to execute should be selected. Segmentation methods with low P and R could be eliminated if they do not have a significant contribution to the quality of the segmentation. Finally, a dynamic parameter choice using the fixed place of the plant (middle of the image) and the background (sides and corners of the image) and their local colour, texture, and edge characteristics could be used to further increase the number of correctly evaluated images. Further, for the edge based segmentation we only used Prewitt based methods as only these were available in the software, but the Canny edge detection procedure might render improved results [24]. Nevertheless, a comparison would be required as the Canny procedure involves a more complex computation and is thus processor/time demanding [24]. Moreover, our current procedure can be further optimized in terms of the number of correctly analyzed images and the processing time needed per image, which now ranges from seconds to tens of seconds.

More sophisticated field-based phenotyping platforms have been developed, such as the tractor-pulled multi-sensor platform BreedVision, that carries a light source allowing exclusion of environmental light [3] could increase throughput of image acquisition. Another option is using unmanned aerial vehicles (UAV), provided that the resolution is sufficient for accurate individual plant identification and characterisation. Both options, however, are expensive and not accessible to most institutes involved in forage crop breeding and research at this moment. Our system has several advantages: it is inexpensive, it has a high-throughput (it takes about 8–10 s to take an image), non-specialised staff can be involved in image acquisition, and standard DSLR cameras are sufficiently robust for common outdoor weather conditions.

### Applications and perspectives

The evaluation of *L. perenne* plants for breeding and selection purposes is typically based on the assignment of visual scores, rendering poor resolution to discriminate differential genotypic responses. Such visual observations are inexpensive but can be biased by the examiner and may not be sufficiently accurate for targeted crop improvement [16, 30]. With the recent developments in high-throughput genotyping for breeding purposes, the demand for quantitative phenotypic data is also increasing, to a point that phenotyping and not genotyping is becoming the bottleneck for genetic improvement of crops [6, 32]. We show here that image analysis-based on-field phenotyping can render quantitative evaluations of growth parameters, probably at the level of resolution required for detailed investigation of the underlying genetic mechanisms and enabling more precise selection in perennial ryegrass.

Although it was not our prime objective to define the best image analysis-derived parameters to quantify (re)growth in perennial ryegrass, the parameters ‘regrowth’, ‘base area’ and ‘first year lateral expansion’, as defined in this work, seem to reproduce quite well the expected overall seasonal responses and the differences between groups of genotypes from different origins. It is therefore possible to use our image-analysis based methodology to describe and follow-up the growth of individual perennial ryegrass genotypes in the field.

Further, our results demonstrate a relatively high correlation between data derived from images and manual measurements. This was the case for the whole collection of 501 genotypes, but also within the different groups considered (breeding forage lines, current forage varieties, current turf varieties and wild accessions). In general, higher correlation values were obtained across cuts and genotypes between ‘base area’ and ‘tiller number’ than between ‘regrowth’ and ‘leaf growth’. In this latter case, top view images as considered here might not be sufficient to capture vigorous leaf elongation of plants with an erect growth pattern. Combination with side-view images or the use of UAV based technologies using digital elevations models (DEMs) could enable to estimate plant height and might render better estimates if the purpose is to obtain reliable information of ‘leaf growth’. Our finding that tiller number can be estimated with a relatively high accuracy from top view images of plants that have been just cut is interesting, as counting tillers is time-consuming and not readily done in practical breeding programs. With our methodology it is therefore possible to get rather good estimates of tillering, which is an important determinant of forage yield and turf quality.

The set of plant parameters derived from image analysis could be extended in the future, using our methodology as start point. Here, we have focused on lateral expansion over a whole season and on growth after cutting over a short period of time (1 week). Growth over a period of a few weeks could be considered but the methodology presented here, in its own, is probably inefficient for the estimation of green biomass accumulation in larger plants, as leaf density increases [12]. As mentioned above, combination of top- and side-view images or UAV derived DEMs could allow estimating the plant biovolume, helping to get accurate estimates of green mass of large plants.

Finally, although not tested here, we anticipate that the high-throughput, inexpensive image analysis procedure presented here can be easily extrapolated to other forage and turf species. In addition, the set of plant traits extracted from the images can be extended in the future, possibly in combination with side-view images able to capture information on leaf density and plant habit (erect or prostrate). This would make the estimation of dry matter accumulation in larger plants possible.

## Conclusion

We have developed a low-cost high-throughput phenotyping system and an image analysis procedure allowing a correct evaluation of 77.2 % of the top view images of field-grown perennial ryegrass plants. It was possible to quantify base area in an objective, quantitative way and to monitor lateral expansion and regrowth during a growing season under field conditions. We demonstrate that the image-derived variables are complementary to manually measured variables such as tiller number and leaf growth. This additional growth describing variables are important for genetic dissection of those traits.

## Additional files

10.1186/s13007-016-0132-8 Cutting regime and measurement periods of the *L. perenne* plants in 2010 (Y1) and 2011 (Y2) related to day of the year (DOY) and growing degree days (GDD^C^, °C day).
10.1186/s13007-016-0132-8 Schematic representation of the image analysis procedure. An overlay image with the composite mask was made to allow for visual inspection. At the same time the selected object was measured and data were exported to a comma-delimited file. After visual inspection for correctness of the overlay image with the composite mask, it was decided if the corresponding measurement data were used or not. Dashed lines indicate the comparisons for the performance testing.
10.1186/s13007-016-0132-8 Relation between leaf growth based and plant height measured at two weeks after cutting per cut and regrowth per group. Linear regressions are fitted and 95 % confidence intervals are shown.
10.1186/s13007-016-0132-8 Relation between tiller number and base area per cut and per group. Linear regressions are fitted and 95 % confidence intervals are shown.
